# Propranolol exhibits activity against hemangiomas independent of beta blockade

**DOI:** 10.1038/s41698-019-0099-9

**Published:** 2019-11-01

**Authors:** Maiko Sasaki, Paula E. North, Justin Elsey, Jeffrey Bubley, Shikha Rao, Yoonhee Jung, Shengnan Wu, Ming-Hui Zou, Brian P. Pollack, Jayanth Kumar, Hartej Singh, Jack L. Arbiser

**Affiliations:** 1 Department of Dermatology, Emory University School of Medicine, Atlanta, GA 30322 USA; 2 Veterans Affairs Medical Center, Decatur, GA 30033 USA; 3 Department of Pathology, Children’s Hospital of Wisconsin, Milwaukee, 53226 USA; 4 Department of Biology, Emory University, Atlanta, GA 30322 USA; 5 Center for Molecular and Translational Medicine, Georgia State University, Atlanta, GA 30303 USA; 6 Stritch School of Medicine, Maywood, IL 60153 USA

**Keywords:** Paediatric cancer, Tumour angiogenesis

## Abstract

Propranolol is a widely used beta blocker that consists of a racemic mixture of R and S stereoisomers. Only the S stereoisomer has significant activity against the beta-adrenergic receptor. A fortuitous clinical observation was made in an infant who received propranolol for cardiac disease, and regression of a hemangioma of infancy was noted. This has led to the widespread use of propranolol for the treatment of large and life-threatening hemangiomas of infancy. Infants receiving propranolol require monitoring to ensure that they do not suffer from side effects related to beta blockade. The exact mechanism of activity of propranolol in hemangioma of infancy is unknown. In this study, we treated hemangioma stem cells with both beta blockade active S- and inactive R-propranolol and looked for genes that were coordinately regulated by this treatment. Among the genes commonly downregulated, Angiopoietin-like 4 (ANGPTL4) was among the most regulated. We confirmed that propranolol isomers downregulated ANGPTL4 in endothelial cells, with greater downregulation of ANGPTL4 using the beta blockade inactive R-propranolol. ANGPTL4 is present in human hemangiomas of infancy. Finally, R-propranolol inhibited the growth of bEnd.3 hemangioma cells in vivo. The implication of this is that hemangioma growth can be blocked without the side effects of beta blockade. Given that humans have been exposed to racemic propranolol for decades and thus to R-propranolol, clinical development of R-propranolol for hemangiomas of infancy and other angiogenic diseases is warranted.

## Introduction

Hemangiomas of infancy represent the most common childhood neoplasm. These lesions occur in up to 10% of infants, with a female predominance and a higher incidence in premature infants.^1^ These lesions have a well-known “life cycle,” characterized by rapid growth, involution, and eventual replacement by a connective tissue scar.^2^ The vast majority of these lesions do not require treatment and can be followed by observation through eventual involution. However, a significant number of these lesions can cause substantial morbidity through compression of vital structures, pain from ulceration, cardiac complications, and scarring. These lesions require systemic therapies. Until recently, systemic agents such as interferon alpha (IFN-α) were used with benefit in conjunction with a high number of side effects.^3^

Several years ago, an infant with a large infantile hemangioma (IH) was administered propranolol to treat cardiac disease. Unexpectedly, the hemangioma regressed. This was followed by a series of patients treated with propranolol, in whom rapid regression was noted.^4^ These results have been replicated at many centers worldwide, and now propranolol is the standard of care for treatment of large and morbid hemangiomas of infancy.^1,5^ Despite the improved treatment of patients with hemangiomas of infancy with propranolol, several unmet needs remain. First, the mechanism of action of propranolol in hemangiomas of infancy is poorly understood. Second, infants receiving propranolol require close monitoring due to the side effects of beta blockade, which include bradycardia and hypoglycemia. Finally, not all hemangiomas respond well to propranolol for unknown reasons.^6^ Propranolol that is administered to patients today consists of a racemic mixture of active (S-propranolol) and inactive (R-propranolol). The R-propranolol is generally assumed to be inactive and is not separated from the S-isomer.^7^

Genetic studies of hemangiomas have not elucidated a common genetic cause, as opposed to other vascular lesions, such as vascular malformations and angiosarcomas, in which genetic mutations have been found.^8–13^ Cytokines, including vascular endothelial growth factor (VEGF) and Angiopoietin-2 (ANG2), have been found in hemangiomas of infancy.^14–16^ The role of these factors in hemangiomas are not fully understood, especially given that ANG2 can serve as a growth factor or mediate endothelial apoptosis depending on the context. Recently, we studied a series of infants who received propranolol for treatment of hemangioma of infancy and found that propranolol reduced VEGF levels but not ANG2 in saliva.^16^ This suggests that propranolol is not acting to decrease ANG2, and also, other angiogenic cytokines might be playing a role. In this study, we treated human endothelial stem cells derived from hemangiomas of infancy with R- and S-propranolol and found that R-propranolol was more effective in downregulating one of the factors in IH. We further investigated the effect of beta blockade negative R-propranolol in a murine model of hemangioma of infancy and found that it is indeed effective in tumor reduction.

One of the genes that was downregulated by all treatments was ANGPTL4. Interestingly, the inactive R-propranolol was far more effective in downregulating ANGPTL4 than the beta blocker active S-propranolol. ANGPTL4 is present in authentic hemangiomas of infancy. Finally, R-propranolol is effective in reducing tumor volume in vivo against bEnd.3 cells, a murine model of hemangioma of infancy.^15,17^ Our results suggest that beta blockade is not required for the effect of propranolol for hemangiomas of infancy. Thus, infants could be treated with propranolol while avoiding the side effects of beta blockade. In addition, potentially higher doses of R-propranolol could be administered to treat hemangiomas and other angiogenic disorders. Finally, we provide the first description of a role of ANGPTL4 in the pathogenesis of hemangioma of infancy.

## Results

### R-isomer of propranolol reduces the expression of ANGPTL4 in hemangiomas of infancy

**Fig. 1:**
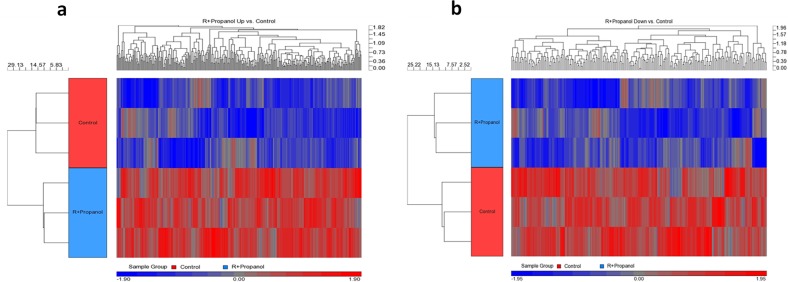
Hierarchical clustering of differentially expressed genes of HemSCs treated with R-propranolol. **a** Heat map of genes that are upregulated in R-propranolol-treated HemSCs. **b** Heat map of genes that are downregulated in R-propranolol-treated HemSC

**Fig. 2:**
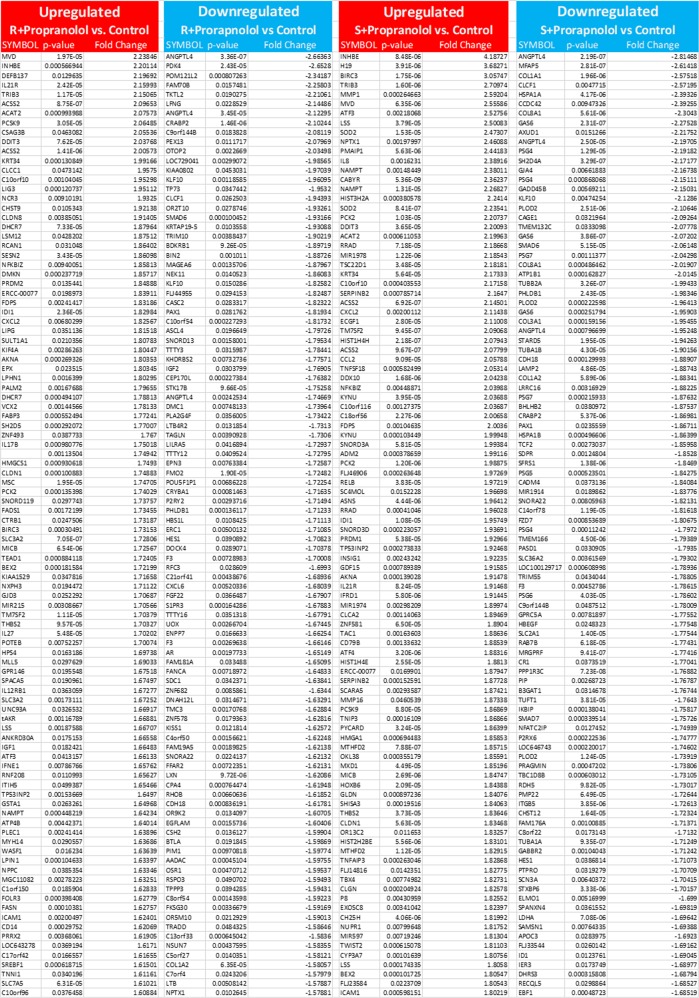
List of 100 most upregulated (red) and downregulated (blue) genes in HemSCs. List of genes upregulated or downregulated in R- or S-propranolol-treated HemSC samples when compared to the vehicle control was sorted by the significance (*p* < 0.05). ANGPTL4 is the most downregulated gene in R-propranolol-treated HemSCs

In order to investigate whether a key gene in hemangiogenesis is regulated by the isomers of propranolol in human hemangiomas of infancy, hemangioma stem cells (HemSCs) were treated with either R- or S-propranolol. HemSCs are human endothelial cells originally isolated from IH, which are known to express glucose transporter 1 (GLUT1^+^) and have been shown to play key roles in hemangiogenesis. Since these cells serve as an in vitro model system for IH, it is of interest to determine whether there are differential effects of R- and S-isomeric components of the widely used racemic propranolol mixture. Angiopoietin-like 4 (ANGPTL4) was found to be one of the most downregulated genes in the R-propranolol-treated group when compared with the cells treated with S-propranolol and control. The data suggest that surprisingly, R-propranolol, not S-propranolol with beta blockade activity, reduces the ANGPTL4 expression in HemSCs (Fig. 1). List of most upregulated and downregulated genes in HemSC samples treated with R- and S-propranolol is appended (Fig. 2).

### Isoforms of propranolol, especially the R-isomer, reduces the expression of ANGPTL4 in bEnd.3 cells as a model of hemangiomas of infancy

Prior to establishing the bEnd.3 in vivo tumor model to study the effects of R-propranolol, ANGPTL4 expression in bEnd.3 cells in vitro was investigated. Furthermore, unlike the benign hemangioma model that spontaneously goes through involution over time, bEnd.3 cells form robust tumor-forming microvascular structures to serve as a target of the therapeutics. Since these cells serve as a mouse model system for IH, it is of interest to determine whether there are differential effects of R- and S-isomeric components of the widely used racemic propranolol mixture.

**Fig. 3:**
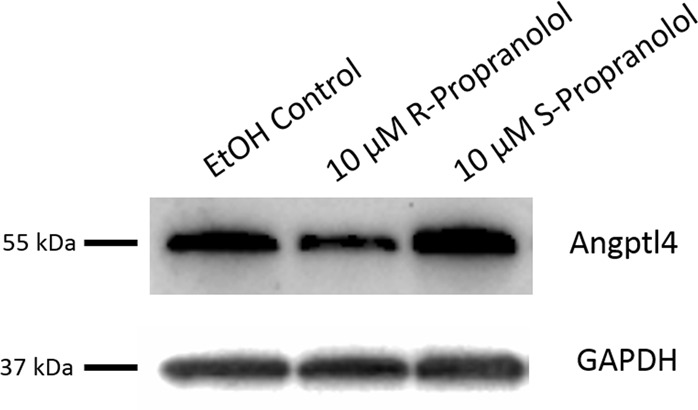
ANGPTL4 protein expression is reduced in R-propranolol-treated bEnd.3 cells. bEnd.3 cells were seeded 24 h prior to the R- or S-propranolol treatment. Culturing media were changed to complete media containing either 10 μM R- or S-propranolol or vehicle control of ethanol. After 24 h propranolol treatment, cells were subjected to western blotting and probed for ANGPTL4. R-propranolol differentially reduced the expression of ANGPTL4 as demonstrated by detection of the 55 kDa expected size bands. Loading control of GAPDH demonstrates that reduction is not due to the loading. Three independent experiments were performed, and a representative image is shown here. Other experimental results from western blotting is described in Supplementary Fig. 2. Titration of R-propranolol ranging from 0 to 10 μM was performed to determine the optimal treatment concentration and described in Supplementary Fig. 3

bEnd.3 was treated with either R- or S-propranolol, and protein lysates were analyzed for ANGPTL4 expression. When compared to the S-propranolol- or vehicle-treated cells, R-propranolol treatment significantly reduced ANGPTL4 expression. Interestingly in S-isomer-treated cells, ANGPTL4 expression was largely unaffected when compared to the vehicle control (Fig. 3). The data suggest that R-propranolol, and surprisingly not the beta blocker S-propranolol, reduces the ANGPTL4 expression in bEnd.3 cells.

### ANGPTL4 is expressed in human hemangioma of infancy

**Fig. 4:**
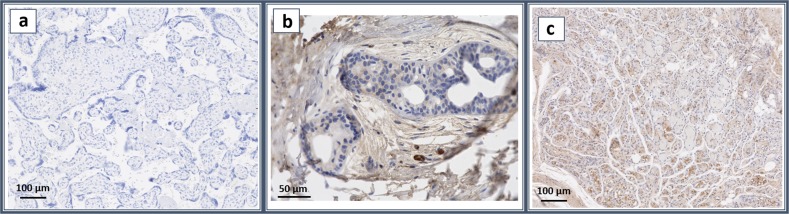
ANGPTL4 is expressed in hemangioma of infancy. Representative images of immunohistochemistry. The panels represent (**a**) no antibody control, (**b**) human infant foreskin sample stained with ANGPTL4, and (**c**) infantile hemangioma sample stained with Angptl4. The scale bars represent 100 μm (**a**, **c**) and 50 μm (**b**). Infantile hemangioma samples highly express ANGPTL4 proteins in endothelial cells, while human foreskin sample consists of largely negative cells surrounded by artifactually stained tissue

To ascertain the physiologic relevance of our findings, ANGPTL4 expression in human IH was examined. Immunohistochemistry of ANGPTL4 was performed on IH tissue obtained from the Pediatric Pathology Department (Children’s Hospital of Wisconsin, Milwaukee). Figure 4 demonstrates that the human hemangioma of infancy tissue does indeed express ANGPTL4 highly in the endothelial cells of the hemangioma. Positive staining of ANGPTL4 in IH (Fig. 4c) is shown against the control tissue without antibody (Fig. 4a) and human infant foreskin stained with ANGPTL4 (Fig. 4b) to illustrate the positive staining. The foreskin sample shows artifactual staining on the tissue surrounding the cells that are negative for staining (Fig. 4b).

### R-propranolol significantly inhibits tumor growth in a preclinical model of hemangioma

**Fig. 5:**
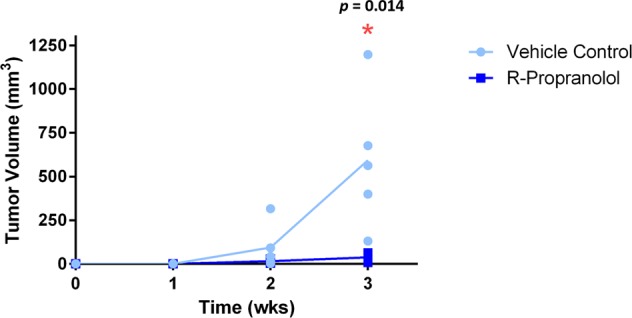
R-propranolol significantly suppressed tumor growth in vivo. bEnd.3 cells allografted into mice formed tumors within 2 weeks of inoculation, and treatment with R-propranolol significantly reduced the tumor volume within 2 weeks of tumor development (*p* = 0.014). Five animals were included in each of the control and the treatment groups, depicted by individual dot in the graph. All statistical analyses were performed using GraphPad Prism (GraphPad Software, La Jolla, CA)

Given that R-propranolol, but not S-propranolol, has efficacy in downregulating ANGPTL4, we evaluated the efficacy of R-propranolol against bEnd.3 cells in vivo. We utilized bEnd.3 cells rather than hemangioma-derived stem cells as the growth of hemangioma-derived stem cells is self-limiting, while bEnd.3 cells exhibit continuous, robust growth. Therefore, inhibition of bEnd.3 growth in vivo is a very stringent test of drug efficacy.^15^ S-propranolol was not tested in this model because (a) the LD50 of S-propranolol in mice is very low due to beta blockade, and (b) S-propranolol did not downregulate ANGPTL4. We found that R-propranolol significantly inhibits the growth of bEnd.3 in vivo, suggesting that R-propranolol, which lacks problematic beta blockade activity, may be sufficiently effective in tumor suppression (Fig. 5).

### RNAseq analysis of R-propranolol- vs. vehicle-treated tumors

**Fig. 6:**
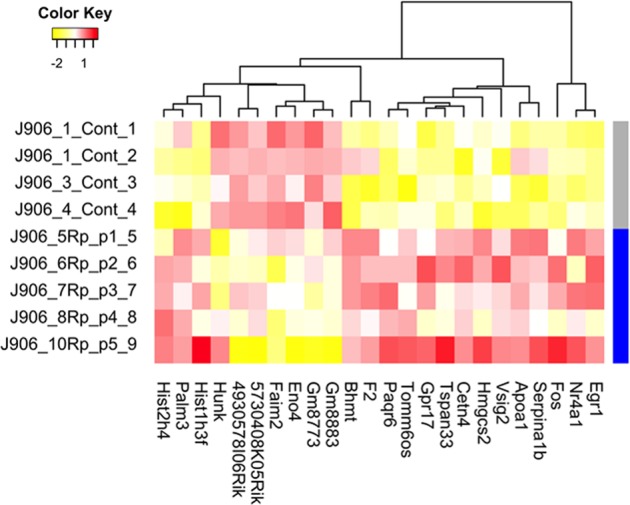
Heat map of differentially expressed RNA between bEnd.3 cells treated with R-propranolol and vehicle. Twenty-four transcripts were identified by RNAseq to be upregulated or downregulated in R-propranolol-treated cells when compared to the vehicle-treated control cells with *p* value ≤ 0.05. Transcripts were further filtered by minimum two-fold difference in the expression levels

**Fig. 7:**
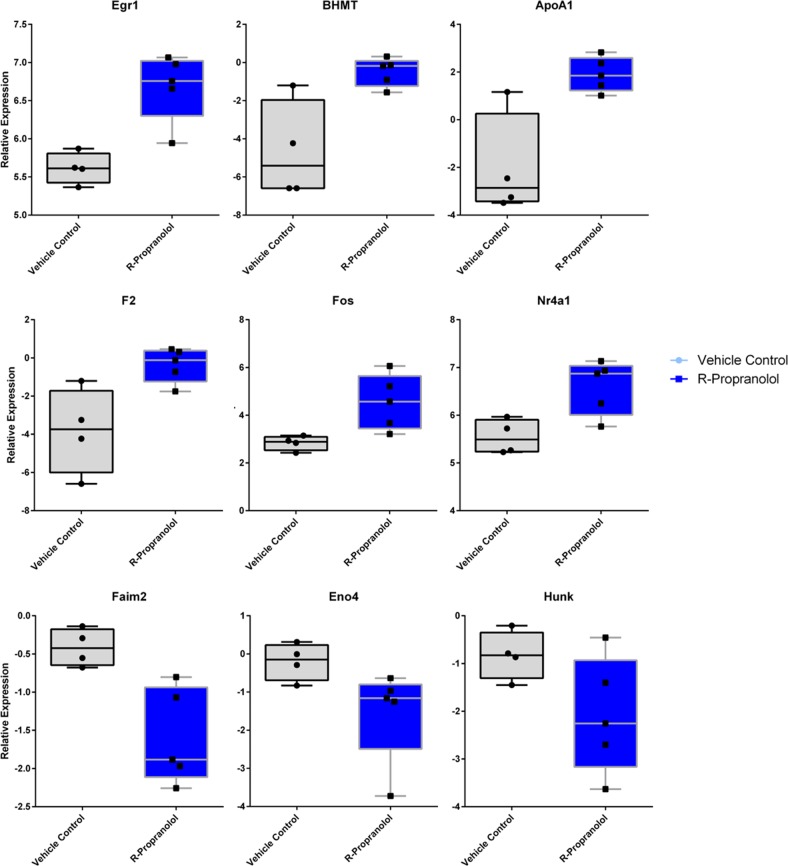
Selection of box plots of differentially expressed RNA transcripts of bEnd.3 cells treated with R-propranolol. Seven transcripts were found to be significantly downregulated, and 17 transcripts were upregulated. Six of the upregulated genes of interest including Egr1, APOA1, and BHMT as well as three of the downregulated genes including Faim2, Hunk, and Eno4 are included for representation. Box plots represent interquartile range with the central line denoting the median, and upper and lower whiskers represent standard error of means. Individual data points are included to demonstrate the spread

In order to gain further insights in the mechanisms of action of R-propranolol in vivo, we subjected vehicle- and R-propranolol-treated tumors to RNAseq analysis. Kyoto Encyclopedia of Genes and Genomes (KEGG) analysis showed that choline metabolism was the pathway most affected by R-propranolol treatment (Figs. 6 and 7).^18–22^ Consistent with this, the most highly regulated gene by R-propranolol treatment was betaine-homocysteine methyltransferase (Bhmt). Interestingly, other genes were involved in tumor-suppressive activities, such as early growth response 1 (Egr1) and AP-1 subunit, which are both transcription factors implicated in tumor suppression.^23,24^ Egr1 regulates important tumor suppressors such as PTEN and p53 and upregulates tumor necrosis factor α. Fos, which has been known to be rearranged and expressed frequently in epithelioid hemangioma, was also upregulated in R-propranolol-treated samples.^25–27^ The genes identified in the RNAseq analysis pose as interesting targets for further investigations.

### Differential gene expression findings from RNAseq analysis were validated in protein expression level in R-propranolol- vs. vehicle-treated tumors in vivo

**Fig. 8:**
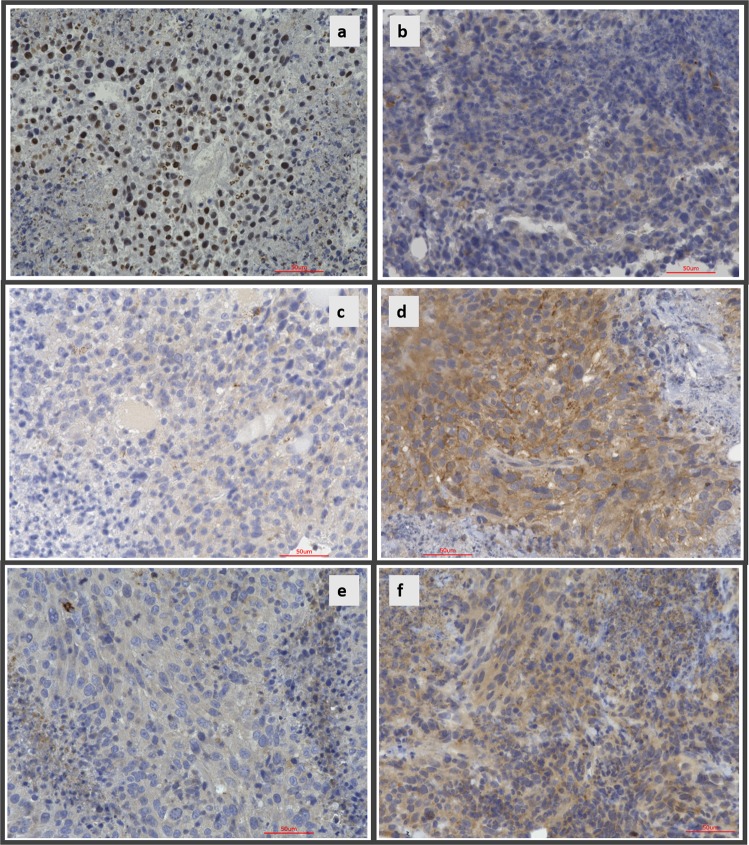
R-propranolol alters changes in the expression of proteins identified in RNAseq, validating the findings. Immunohistochemistry for ANGPTL4, BHMT, and APOA1 were performed on paraffin-embedded samples of R-propranolol- or ethanol vehicle-treated bEnd.3 murine tumor to validate the differential expression analysis results obtained using RNAseq. Nuclear expression of ANGPTL4 was markedly reduced in R-propranolol-treated animals while BHMT and APOA1 expression was increased in the experimental group, supporting the RNAseq findings. **a**, **b** Control- and R-propranolol-treated tumor samples stained with ANGPTL4. **c**, **d** Control- and R-propranolol-treated tumor samples stained with BHMT. **e**, **f** Control- and R-propranolol-treated tumor samples stained with APOA1. Scale bars indicate 50 μm in all panels

To investigate whether differential expression of genes identified in RNAseq analysis was observed at the protein expression level in the murine tumor system, immunohistochemistry on three of the identified key genes, ANGPTL4, BHMT, and APOA1, was performed (Fig. 8). In accordance with the RNAseq findings,^28^ nuclear ANGPTL4 expression was strongly reduced in R-propranolol-treated animals (Fig. 8b) when compared to the vehicle control animal (Fig. 8a). BHMT and APOA1, which were found to be greatly induced in R-propranolol-treated animals, also showed increased cytosolic expression in vivo (Fig. 8b–e), indicating that the changes detected are at the transcription as well as the translational level.

## Discussion

Hemangiomas of infancy are the most common tumor of childhood and have not consistently been associated with a specific mutation, despite being clonal. Signaling abnormalities have been described in hemangiomas of infancy, including Glut-1 expression, cytoplasmic WT-1 expression, and elevated levels of NADPH oxidase.^29^ While most hemangiomas do not require treatment, a significant subset of hemangiomas causes significant and even life threatening consequences, including compression of the trachea, ocular damage, and disfigurement.^2^ Hemangiomas are also associated with PHACE syndrome, in which hemangiomas are associated with other abnormalities, including posterior fossa brain malformations, and cardiac abnormalities.^1,30^ The fortuitous discovery of propranolol causing regression of hemangiomas has revolutionized the treatment of these lesions.^4^ However, treatment of hemangiomas with propranolol is not risk free because propranolol may cause bradycardia, hypotension, and hypoglycemia as a consequence of beta blockade.^5,6^ While the presence of beta adrenergic receptors has been recognized on hemangioma endothelium, the role of beta blockade as the mechanism of hemangioma regression has not been established.

We hypothesized that propranolol works through beta blockade-independent mechanisms. Commercial propranolol is a mixture of S-propranolol (beta blocker) and R-propranolol (non-beta blocker). The same is true for other commercially available beta blockers, which are synthesized as aryl ethers of epichlorohydrin and then reacted with a primary amine, leading to an optically active center, which is sold as a racemic mixture based on the assumption that the R-isomer is biologically inactive. We used purified isomers of propranolol to assess the validity of this hypothesis. We treated HemSCs with R- and S-propranolol. Another gene that was most coordinately regulated by this treatment was ANGPTL4, which was downregulated by all three treatments on gene array. We examined the expression of ANGPTL4 protein and showed that the R-isomer (non-beta blocker) suppressed ANGPTL4, but the beta blocker S-isomer had no effect. Using immunohistochemistry, we demonstrated that ANGPTL4 is present in authentic hemangioma of infancy.

Because R-propranolol appeared to have greater activity and S-propranolol has a low LD50 in mice due to beta blockade, we assessed the ability of R-propranolol to block the growth of bEnd.3 hemangioma in vivo. bEnd.3 is a useful preclinical model that recapitulates many of the signaling abnormalities of hemangioma of infancy and is useful for in vivo studies as human hemangioma endothelia do not grow robustly in mice.^15,17,31,32^ R-propranolol caused significant inhibition of tumor growth. We performed RNAseq analysis of treated tumors against vehicle tumors and found several unexpected findings. The gene that was most upregulated by R-propranolol treatment was BHMT, and this enzyme converts toxic homocysteine into methionine using betaine as a methyl donor.^33,34^ KEGG analysis showed that choline metabolism was highly affected. Egr-1, an AP-1 subunit with tumor-suppressive activity, was also elevated by R-propranolol treatment. APOA1 (high-density lipoprotein), which protects against vascular instability was also induced by R-propranolol. N4RA1 (Nur77) is also upregulated by propranolol and is associated with tumor suppression in hepatocellular carcinoma by suppressing glycolysis.^35,36^

Hemangioma of infancy is widely known as a glycolytic tumor indicated by (a) [F-18] fluoro-deoxyglucose uptake detected in positron emission tomography and (b) GLUT-1 expression, which promotes glucose uptake and glycolysis. To investigate the potential role of propranolol isomers in IH, we examined the effect of propranolol isoforms on metabolism in bEnd.3 hemangioma cells indicated by reduction via changing the oxidative status of tumor cells. Mitochondrial respiratory capacity was measured using the Seahorse XF Cell Mito Stress Test Kit, which is a standard metric in measuring oxidative functions of mitochondria. Both isomers of propranolol converted bEnd.3 cells to a respiratory phenotype as indicated by the increase in maximal respiration (Supplementary Fig. 4a) as well as the drastic increase in basal respiration (Supplementary Fig. 4b). Increased spare respiratory capacity of cells treated by either isomer of propranolol also indicated that the cellular survival was promoted in conditions under cellular stress in cells treated with propranolols (Supplementary Fig. 4b).

**Fig. 9:**
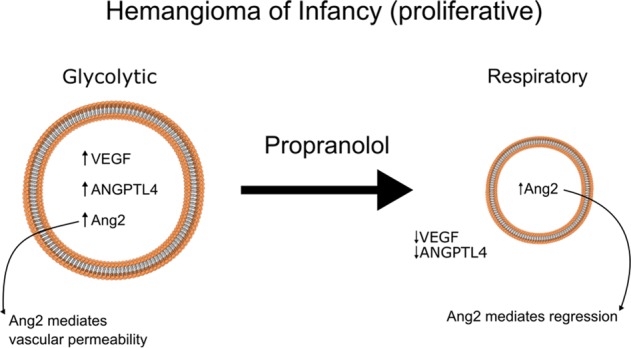
Proposed mechanism of action of propranolol in hemangioma of infancy. Proliferative hemangiomas are glycolytic and have elevated levels of VEGF, Ang2, and ANGPTL4, resulting in a highly angiogenic tumor with leaky vessels. Treatment with propranolol results in conversion to a respiratory phenotype, with downregulation of VEGF and ANGPTL4, but retaining Ang2, which then mediates regression of hemangiomas in the absence of angiogenic stimulation

Given these studies and our previous clinical data showing that propranolol downregulates VEGF but not ANG2 in children, we can express a mechanism of action for the treatment of IH by propranolol. Propranolol causes a coordinated downregulation of VEGF and ANGPTL4 but not Ang2.^16^ The coordinated downregulation of VEGF and ANGPTL4 allows Ang2 to mediate vascular regression (Fig. 9), as Ang2 causes endothelial apoptosis in the absence of VEGF^37^ and perhaps ANGPTL4.^38–40^ Given that these angiogenic cytokines are present in other tumors as well,^41,42^ this may explain the cancer-protective effect of propranolol. Further studies of R-propranolol in humans are warranted.

## Methods

### Cell lines and reagents

Human HemSCs were kindly provided by Dr. Joyce Bischoff (Boston Children’s Hospital, Harvard Medical School). bEnd.3 cells, murine middle T-antigen-transformed endothelial cells derived from endothelioma, were purchased from American Type Culture Collection (Manassas, VA). High-glucose Dulbecco’s Modified Eagle Media (HGDMEM), trypsin-EDTA, and Dulbecco’s phosphate-buffered saline (DPBS) were purchased from Sigma-Aldrich (St. Louis, MO). ANGPTL4 antibody for western blotting and immunohistochemistry were purchased from Thermo Fisher Scientific (# 701155) (Waltham, MA) and Abcam (#ab196746) (Cambridge, UK) respectively. BHMT (#ab36415) and APOA1 (#ab227455) primary antibodies were obtained also from Abcam (Cambridge, UK). Glyceraldehyde 3-phosphate dehydrogenase (GAPDH; #MAB371) antibody was purchased from Millipore (Burlington, MA). Goat anti-mouse secondary antibody conjugated with horseradish peroxidase (HRP; #115-032-003) was purchased from Jackson ImmunoResearch Laboratories (West Grove, PA). Goat anti-rabbit secondary antibody conjugated with HRP (#7074) was purchased from Cell Signaling Technologies (Danvers, MA).

### Cell culture

bEnd.3 was maintained in HGDMEM supplemented with 10% fetal bovine serum (Atlanta Biologicals, Atlanta, GA), 4 mM glutamine, 100 U/mL penicillin and 100 µg/mL streptomycin (Sigma-Aldrich, St. Louis, MO), and 10 ng/mL mVEGF (Cell Signaling Technology, Danvers, MA) in an atmosphere of 37 °C with 5% CO_2_. HemSC was maintained in Endothelial Growth Media 2 supplemented with 2% fetal calf serum and complete EGM supplements (PromoCells, Heidelberg, Germany) under a 5% CO_2_ atmosphere. HemSC was dissociated using 0.05% Trypsin-EDTA (Sigma-Aldrich) and subcultured into flasks precoated with 0.1 μg/cm^2^ fibronectin (Millipore) in 0.1 M Na_2_CO_3_, pH 9.4. Media for all cell lines were changed every 2 days.

### Western blot

bEnd.3 cells grown in T75 flasks were lysed in Pierce RIPA buffer [25 mM Tris-HCl, 150 mM NaCl, 1% NP-40, 1% sodium deoxycholate, 0.1% sodium dodecyl sulfate, pH 7.6] (Thermo Fisher Scientific) supplemented with HALT protease phosphatase inhibitor cocktails. Lysates were centrifuged at 16,000 × *g* for 20 min after 30-min incubation on ice. Protein concentration was determined using BioSpectrometer (Eppendorf, Hamburg, Germany) and diluted in 4× LDS sample buffer (Thermo Fisher Scientific). 30 µg of samples were loaded onto NuPAGE 4–15% precast gels in MOPS buffer (Thermo Fisher Scientific) against Precision Plus Protein Dual Color Standards (Bio-Rad Laboratories). Proteins were transferred onto polyvinylidene difluoride membrane using Transblot Turbo system (Bio-Rad Laboratories, Hercules, CA). Membrane was blocked for 1 h at room temperature in 5% non-fat dry milk in 0.1% Tween-Tris-Buffered Saline and probed with ANGPTL4 or GAPDH antibody at 1:1000 or 1:2000 dilution respectively, at 4 °C overnight. Antibody signal was detected using SuperSignal West Femto chemiluminescence substrate (Thermo Fisher Scientific) and ChemiDoc XRS Gel Photo Documentation system (Bio-Rad Laboratories). Images were processed using the ImageLab software (Bio-Rad Laboratories). All blots derived from the same experiment were processed in parallel, and images were not spliced or merged from other experiments, shown in entirety as well as overlaid with the visible light images to indicate the location of the ladder (see Supplementary Fig. 1). Three independent experiments were performed, and a representative image is shown here. Other experimental results from western blotting are described in Supplementary Fig. 2. Titration of R-propranolol ranging from 0 to 10 μM was performed to determine the optimal treatment concentration and described in Supplementary Fig. 3.

### In vivo bEnd.3 allograft model

The allograft model was developed and approved by the Institutional Animal Care and Use Committee (IACUC) of Emory University, and all methods were performed in accordance with the approved IACUC protocol guidelines and regulations. bEnd.3 cell suspension in growth medium was inoculated at 2.5 × 10^5^ cells/mouse in the right flank of athymic Nu/Nu nude male mice (*n* = 5 per group) purchased from the Charles River Laboratories (Wilmington, MA). R-propranolol was prepared as a 10 mM concentration stock in ethanol and was further diluted to the prescribed final concentration in DPBS. Vehicle control (ethanol in DPBS) or R-propranolol was administered intraperitoneally five times a week at 6 mg/kg/day. R-propranolol treatment was initiated on the second day after the tumor cell injection, and the tumor volume as well as the weight of the animals were recorded weekly thereafter.

### Statistical analysis

The statistical analysis of tumor volumes was performed as previously described, and statistical analyses were performed using the Microsoft Office Excel (Microsoft Corporation, Richmond, WA) and GraphPad Prism software (GraphPad Software, La Jolla, CA).^3^ Briefly, tumor volume was calculated using the formula, volume = (*L* × *W*^2^) × 0.52, where *L* was defined to be the longer dimension of the tumor. Replicate size per group was 5, and unpaired two-tailed Student’s *t*-test was performed to determine the significant difference between the two groups, with significance determined at *p* < 0.05. The values were exported to GraphPad Prism to obtain plots including individual data points to indicate the general spread of the data.

### RNA extraction

Previously frozen tumor tissue was lysed and homogenized in QIAzol® using a rotor-stator probe homogenizer until fully disrupted. RNA extraction was performed using the Qiagen miRNeasy Kit with on-column DNase treatment according to the manufacturer’s specifications (Qiagen, Hilden, Germany). The concentration of RNA eluted in nuclease-free water was determined using a NanoDrop 1000 (Thermo Fisher Scientific). RNA was analyzed on Agilent 2100 Bioanalyzer (Agilent Technologies, Santa Clara, CA), using RNA 6000 Nano assay for quality control prior to RNAseq. Two hundred and fifty nanograms of total RNA was amplified and labeled using the Thermo Fisher Scientific Illumina™ TotalPrep™ RNA Amplification Kit (Thermo Fisher, Waltham, MA) according to the manufacturer’s protocol. Labeled cRNA was hybridized to Illumina HT12 bead array according to the protocol described in the WGGEX Direct Hybridization Assay user guide. Image acquisition and data extraction were performed with an Illumina HiScan laser scanner and GenomeStudio software (Illumina, San Diego, CA).

### Gene array of HemSCs

HemSC was treated with propranolol. Two hundred and fifty nanograms of total RNA, extracted and quality controlled as above, was amplified and labeled using the Thermo Fisher Scientific Illumina™ TotalPrep™ RNA Amplification Kit according to the manufacturer’s protocol. Labeled cRNA was hybridized to Illumina HT12 bead array according to the protocol described in the WGGEX Direct Hybridization Assay user guide. Image acquisition and data extraction were performed with an Illumina HiScan laser scanner and GenomeStudio software.

### RNAseq (bulk) data analysis

RNAseq library preparation was performed at Novogene Corporation utilizing the NEBNext Ultra RNA Library Prep Kit for Illumina by following the manufacturer’s recommendations (New England Biolabs, Ipswich, MA). Sequencing libraries were validated on the Agilent TapeStation (Agilent Technologies, Santa Clara, CA) and quantified using Qubit 2.0 Fluorometer (Thermo Fisher Scientific, Waltham, MA) as well as by quantitative PCR (Applied Biosystems, Foster City, CA). The libraries were sequenced on an Illumina sequencer using a 2 × 150 Paired End configuration. Raw sequence data (.bcl files) was converted into *fastq* files and de-multiplexed using the Illumina’s bcl2fastq software.

### Immunohistochemistry of hemangioma of infancy

Formalin-fixed, paraffin-embedded hemangioma tissue was obtained from the Pediatric Pathology Department (Children’s Hospital of Wisconsin, Milwaukee) following the institutional review board-approved protocol. Tissue sections were cut at 4 µm thickness, deparaffinized, and blocked with peroxidase block and serum-free protein block (Dako, Agilent Technologies). The slides were incubated with the primary ANGPTL4 antibody at a concentration of 1:20, followed by secondary antibody. The stains were visualized with DAB (Dako) and counterstained with hematoxylin. Detection was performed with MACH2 Universal HRP (Biocare Medical, Pacheco, CA). Human infant foreskin sample was resected and embedded in formalin, processed, and stained at Winship Cancer Institute Research Histology Core laboratory (Atlanta, GA). The slides were incubated with the primary antibody, ANGPTL4, at 1:100 concentration followed by secondary antibody. Detection was performed using Keyence BZ-X800 (Keyence, Osaka, Japan).

### Immunohistochemistry of bEnd.3 murine tumor model

Tumor samples were resected and embedded in formalin, processed, and stained at Winship Cancer Institute Research Histology Core laboratory (Atlanta, GA). The slides were incubated with the primary antibodies ANGPTL4, BHMT, and APOA1 at concentrations 1:100, 1:800, and 1:400 respectively, followed by secondary antibody. Detection was performed using Keyence BZ-X800 (Keyence, Osaka, Japan).

### Measurement of oxygen consumption rate (OCR)

OCR was measured using an XF^e^96 extracellular flux analyzer (Seahorse Biosciences, North Billerica, MA). Briefly, bEnd.3 cells were seeded at density of 10,000 cells/well in a 96-well seahorse cell culture plate (#102416-100, Seahorse Biosciences, North Billerica, MA) in DMEM and treated with vehicle or propranolol for 24 h. Proper cell seeding density was screened and adjusted to approximately 100 pmol/min for basal OCR for the control group. Carbonyl cyanide *p*-(tri-fluromethoxy)phenyl-hydrazone (FCCP) titration assay was applied to determine the optimal FCCP dosage (2 µmol/L) by continuously injecting FCCP until the OCR value decreased. Before the measurement, the medium was replaced with XF assay medium (#102365-100, Seahorse Biosciences, North Billerica, MA) containing 10 mmol/L glucose, 1 mmol/L pyruvate, and 2 mmol/L glutamine at pH 7.4 and incubated in a non-CO_2_ incubator at 37 °C for at least 30 min. Stock solutions of oligomycin, FCCP, and rotenone/antimycin A in an XF Cell Mito Stress Test Kit (#103015-100, Seahorse Biosciences, North Billerica, MA) were prepared in XF assay medium and loaded into injection ports A, B, and C, respectively. The working concentrations for each inhibitor were as follows: oligomycin 1 µmol/L, FCCP 2 µmol/L, rotenone 0.5 µmol/L, and antimycin A 0.5 µmol/L. Measurements were obtained at 37 °C. Assay cycles included 3 min of mixing, 2 min of a waiting period, and 3 min of measurement. The *Wave* software provided by Seahorse Biosciences was used for data collection. Basal respiration, maximal respiration, proton leak, and coupled respiration, as well as other measurement parameters were determined using the *XF Cell Mito Stress Test Generator* software (North Billerica, MA).

### Reporting summary

Further information on research design is available in the Nature Research Reporting Summary linked to this article.

## Supplementary information

Supplementary Information

Reporting Summary

## Data Availability

All the data generated and analyzed during the current study are available from the corresponding author on reasonable request.
