# Supplementary material for: Propranolol exhibits activity against hemangiomas independent of beta blockade
Source: NPJ Precis Oncol. 2019 Nov 1;3:27. doi: 10.1038/s41698-019-0099-9 (PMC6825155; doi:10.1038/s41698-019-0099-9)
Supplement: Supplementary file 1 — Supplementary Information [file 41698_2019_99_MOESM1_ESM.pdf]

Supplemental Figures

Propranolol exhibits activity against hemangiomas independent of beta blockade

Maiko Sasaki[1,2], Paula North[3], Justin Elsey[1], Jeffrey Bubley[1], Shikha Rao[1], Yoonhee Jung[4], Shengnan Wu[5], Ming-Hui Zou[5], Brian P. Pollack[1, 2], Jayanth Kumar[6], Hartej Singh[1], Jack L. Arbiser[1,2]

Department of Dermatology, Emory University School of Medicine, Atlanta, GA, 30322[1], Veterans Affairs Medical Center, Decatur, GA 30033[2], Department of Pathology and Laboratory Medicine Children's Hospital of Wisconsin, 53226[3], Department of Biology, Emory University, Atlanta, GA 30322[4], Center for Molecular and Translational Medicine, Georgia State University, Atlanta, GA, 30303[5], Stritch School of Medicine, Maywood, IL, 60153[6].

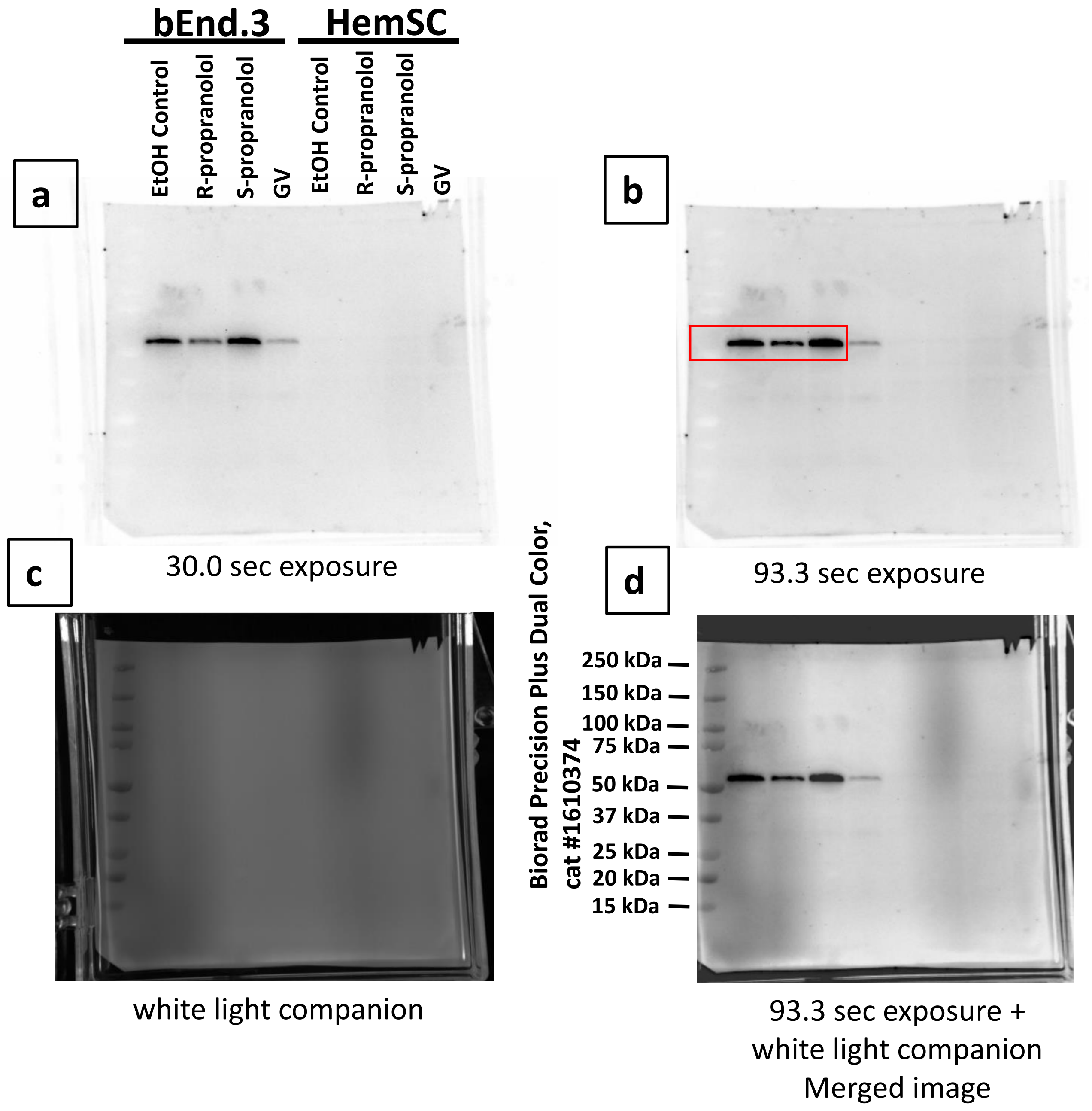

Supplemental Figure 1a. **Image integrity for ANGPTL Western blot** : Western blot images are disclosed in entirety with representative multiple exposures. Standard information used in the Western blot is also included. The lanes and bands used for the figures in the manuscript text are indicated in red boxes. a) 30.0 sec exposure, b) 93.3 second exposure, c) visible light image of the blot, and d) merged image of the Western blot and visible light image to indicate the location of the standards with the bands detected by the antibody.

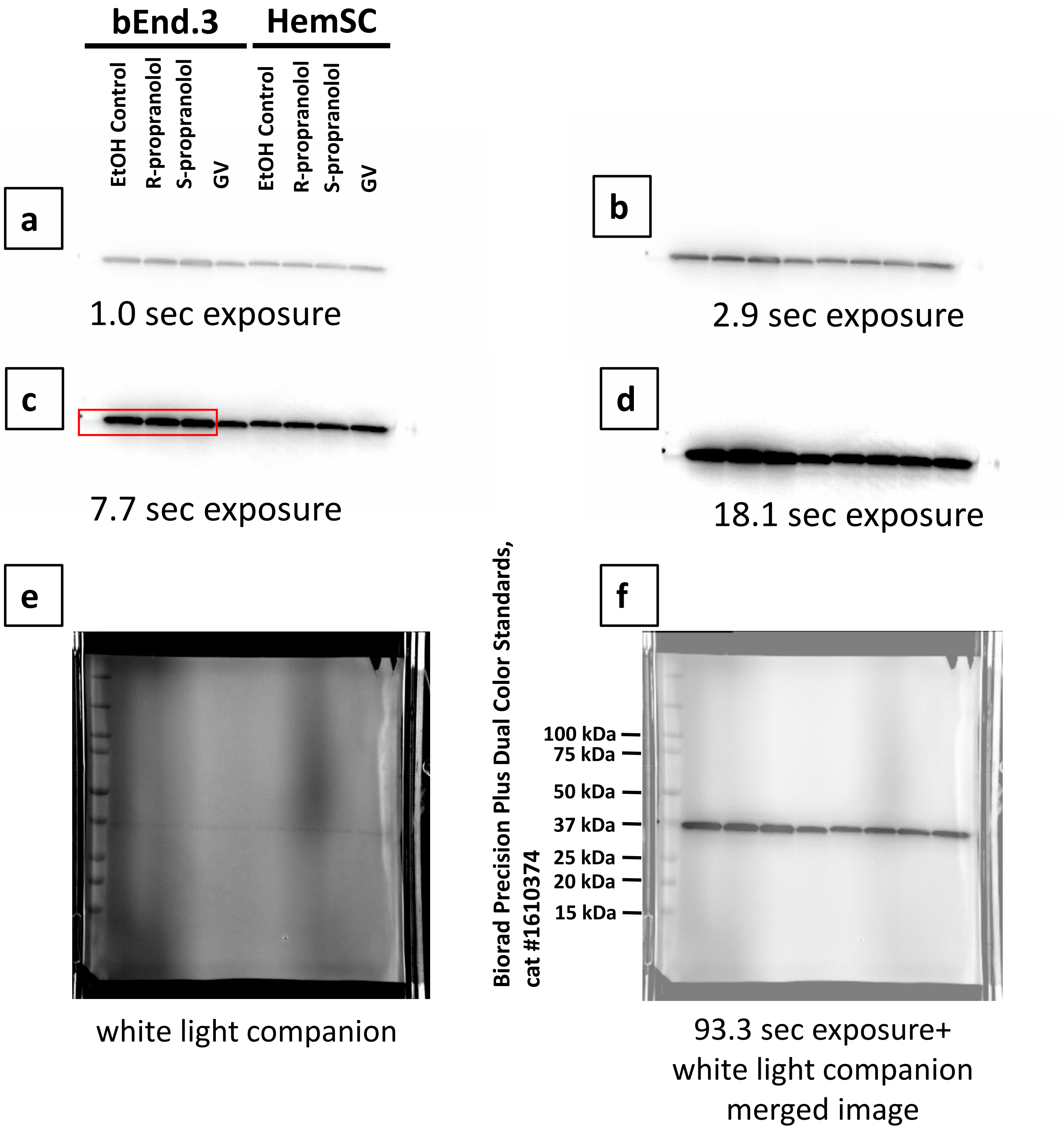

Supplemental Figure 1b. I**mage integrity for GAPDH Western blot** : Western blot images are disclosed in entirety with representative multiple exposures. ladder information used in the Western blot is also included. The lanes and bands used for the figures in the manuscript text are indicated in red boxes. a) 1.0 sec exposure, b) 2.9 second exposure, c) 7.7 sec exposure indicating the samples represented in the manuscript shown in red box, d) 18.1 sec exposure, e) visible light image of the blot, and f) merged image of the Western blot and visible light image to indicate the location of the standards with the bands detected by the antibody.

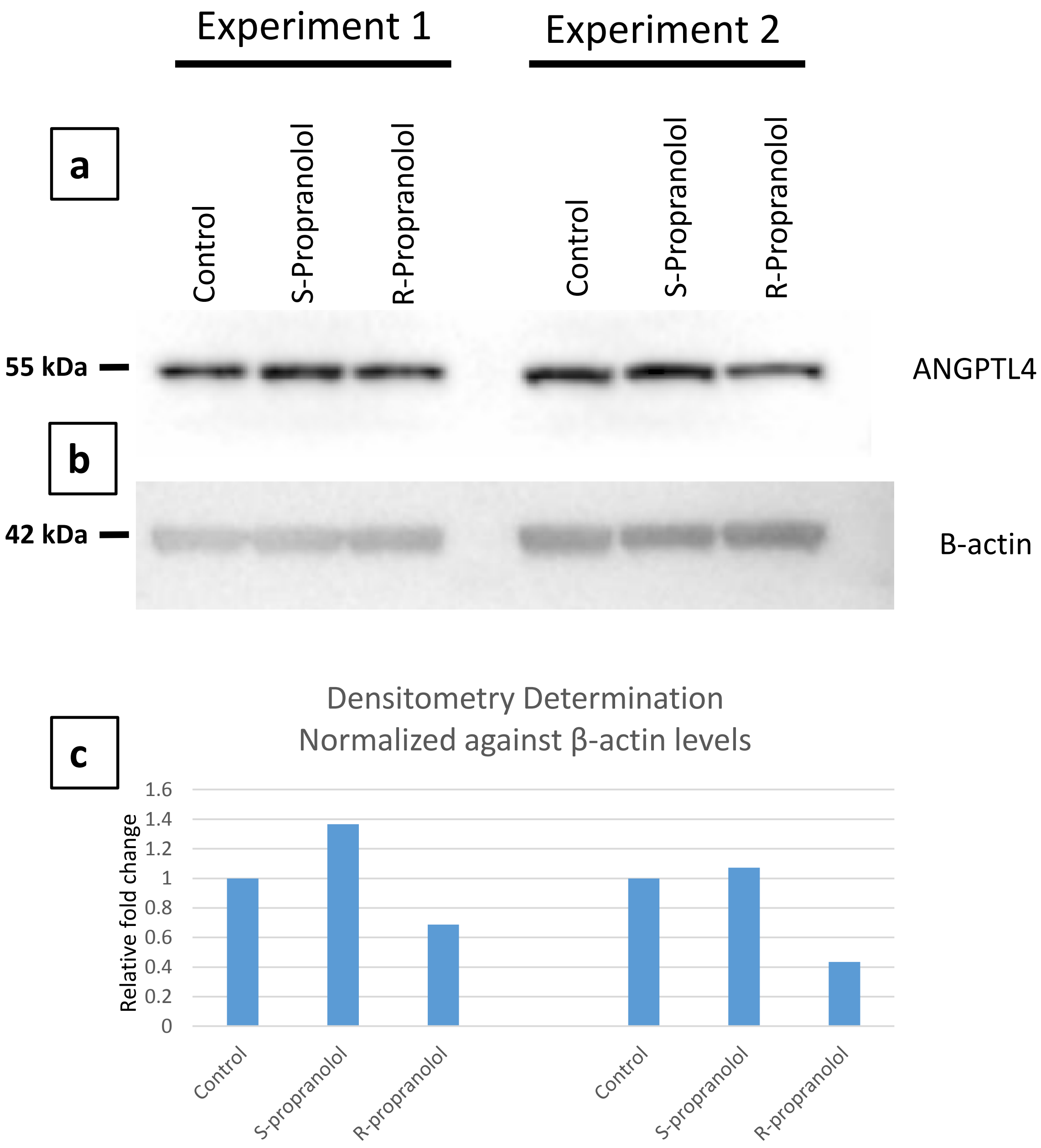

Supplemental Figure 2. **Replicate samples of ANGPTL4 Western blots**. Two replicates out of three independent experiments showing a) ANGPTL4 and b) β-actin are represented here. Third experiment is represented in the main article (Figure 3). Densitometry analyses were performed, and the target bands of ANGPTL4 were normalized against the house keeping protein, β-actin (c) (Cell Signaling Technology, Cat.# 3700S, 1:2,000) .

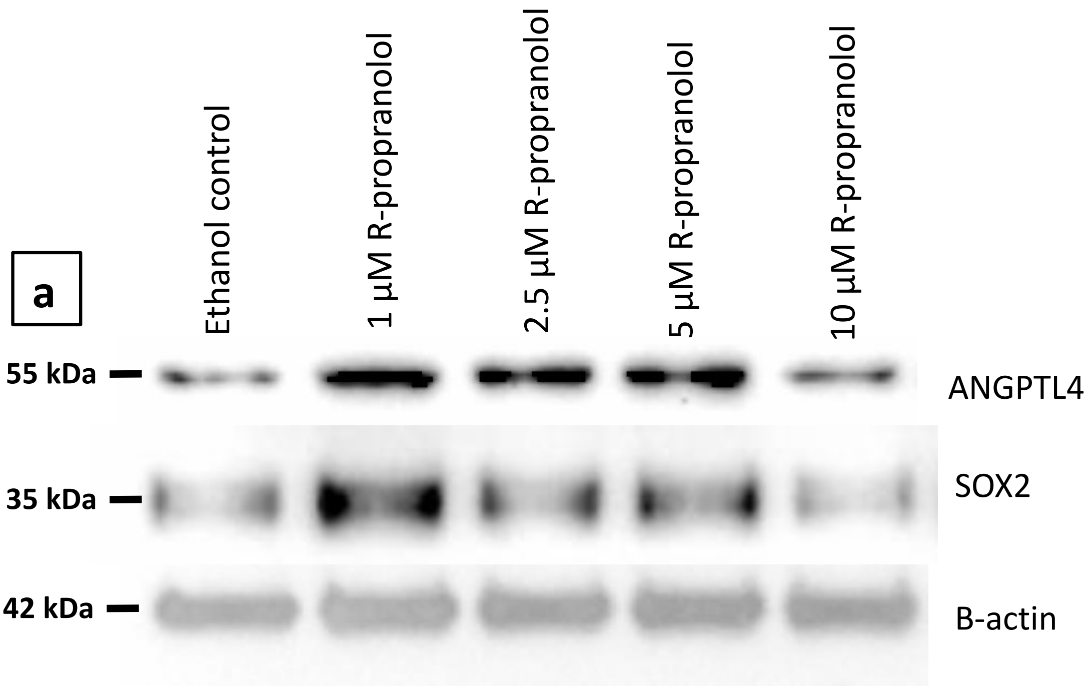

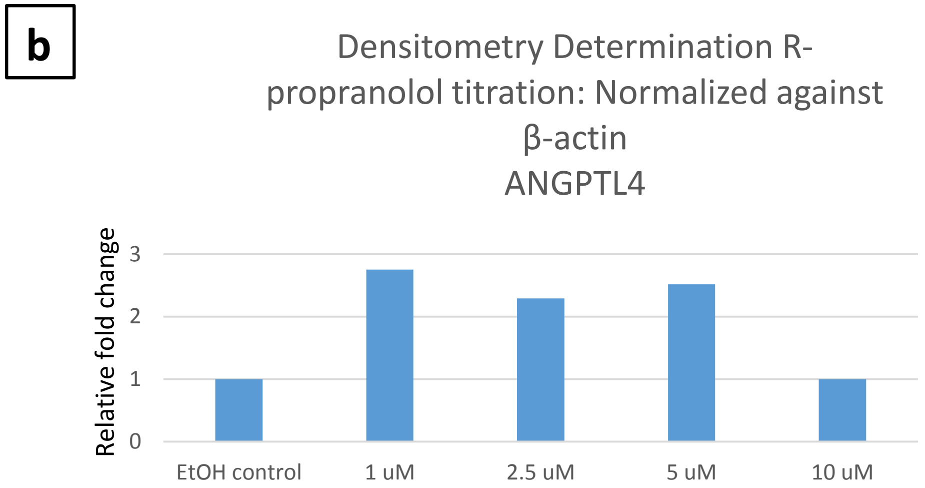

Supplemental Figure 3. **Western blot of R-propranolol titration samples for treatment optimization.** Optimal treatment concentration for both in vivo and in vitro analyses was determined by titrating the R-propranolol concentration ranging from 1 to 10 µM in ethanol by Western blot. A) 10 µM R-propranolol treatment decreased ANGPTL4 and SOX2 expression without affecting cell viability (not shown) and β-actin expression. B) Densitometry analyses were performed, and the target bands of ANGPTL4 were normalized against the house keeping protein, β-actin. SOX2 expression is also shown (Abcam, Cat. #ab171380, 1:1,000).

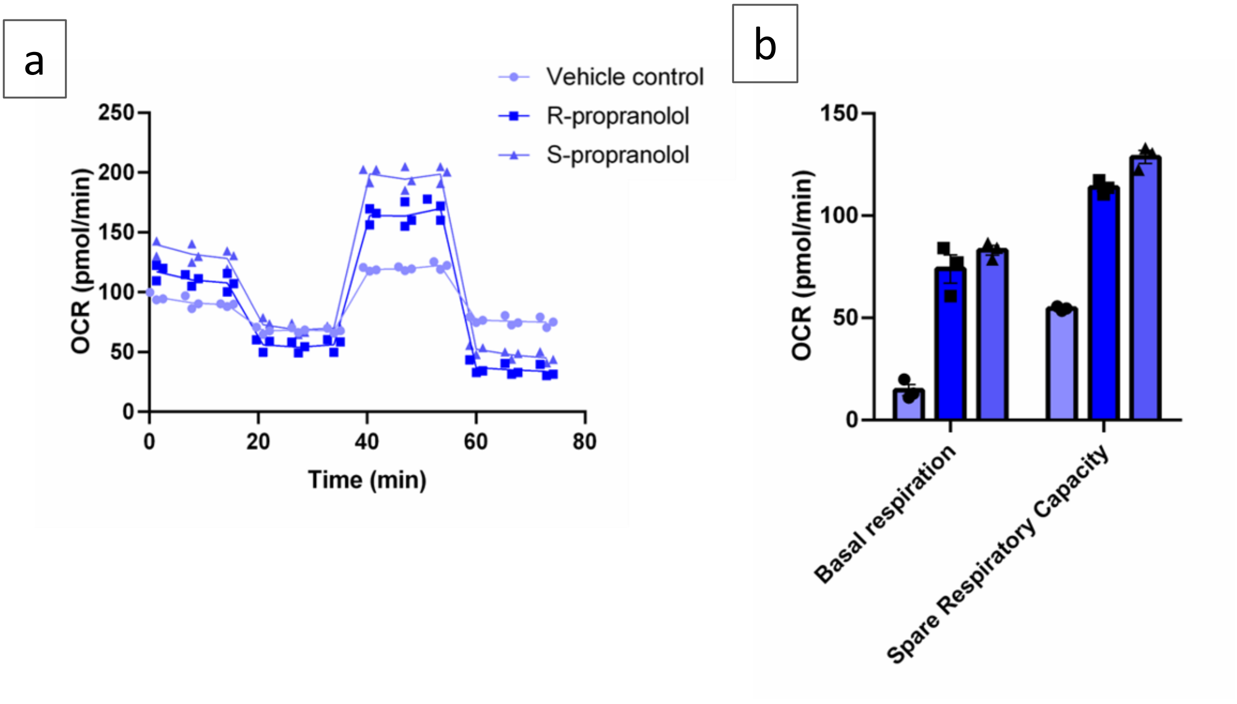

Supplemental Figure 4: R- and S-propranolol induce metabolic changes towards respiration irrespective of beta blockade. R- and S-propranolol treated bEnd.3 cells were assayed with serial injection of oligomycin, FCCP, and combination of rotenone and antimycin A to measure ATP production, maximal respiration, and nonmitochondrial respiration respectively. The spare respiratory capacity as well as proton leakage are calculated from these measurements. a) Both R- and S-propranolol treatments substantially increased maximal respiration compared to the vehicle control. R-propranolol, however, induced stronger respiration than S-propranolol. b) R- and S-propranolol increased basal respiration as well as the spare respiratory capacity.
